# Emergency response to nuclear, biological and chemical incidents: challenges and countermeasures

**DOI:** 10.1186/s40779-015-0044-3

**Published:** 2015-09-07

**Authors:** Hai-Long Li, Wen-Jun Tang, Ya-Kun Ma, Ji-Min Jia, Rong-Li Dang, Er-Chen Qiu

**Affiliations:** Center for Disease Control and Prevention of Xinjiang Military Command, Urumqi, 830011 China

**Keywords:** Medical rescue, Nuclear, Biological and chemical incidents

## Abstract

Given the multiple terrorist attacks that have occurred in recent years in China, medical rescue teams and specialized incident assessment teams have been established by the government; however, medical rescue after nuclear, biological, and chemical incidents remains challenging and is often inefficient. In the present article, problems were analyzed regarding the assessment of responder countermeasures, training of professionals and the management of emergency medical incidents related to nuclear, biological and chemical attacks. Countermeasures, the establishment of response coordination, public education, practical training and exercise, and a professional consultant team or system should be the focus of emergency medical response facilities. Moreover, the government was offered professionals who are involved in managing nuclear, biological and chemical incidents.

## Background

Emergency medical rescue after nuclear, biological and chemical (NBC) incidents has been an increasing concern of many governments since the sarin attack in a Tokyo subway in 1995 [1], the anthrax mail incidents after September 11, 2001 [2], and the stabbings that occurred in Urumqi, China, after July 5, 2009. The threat of exposure of the civilian population to nuclear, biological, and chemical agents, which has traditionally been considered a military issue, has increased due to the potential causes from direct military attacks, stolen weapons of mass destruction (WMDs), acts of terrorism, and industrial accidents and disasters. Based on mass casualty effects, the release of such NBC agents might create thousands of casualties, thereby overwhelming local health and medical resources. Consequently, national medical rescue teams for responding to NBC incidents were established to provide emergency medical services, such as incident assessment, the detection and identification of the agents, on-site treatment and triage, and decontamination of contaminated areas and victims exposed to NBC agents. Again, it is critical that the government addresses both the emergency medical and emergency health issues during medical preparedness and response operations and the system of cooperation between the relevant organizations. The present article analyzed the challenges in the face of NBC incidents in Xinjiang and the emergency medical and emergency public health issues related to medical preparedness and response. This analysis is followed by a preliminary discussion of the corresponding countermeasures.

### Challenges of NBC incidents in Xinjiang

Recently, several violent terrorist attacks launched by Xinjiang terrorists produced hundreds of victims including women and children, which demonstrated that the anti-terror initiatives in Xinjiang are challenging. Threats involving NBC not only come from internal terror groups but also external terrorists: a. the stabbing terror case on July 5, 2009, which was proved to be a non-biological incident, indicated the potential start of a NBC attack by the Three Force terrorist group; b. there are almost one hundred civilian and military factories or facilities in Urumqi Xinjiang that contain large amounts of nuclear, biological, and chemical materials, which can be easily access by terrorists through theft or illegal purchasing, or even through an unintentional leak, which could trigger a NBC accident, such as the Yuantong poison mail case in November, 2013 and the radioactive source loss in Nanjing in May, 2014; c. countries neighboring Xinjiang, which inherited some NBC weapons from the Soviet Union after the collapse, are almost entirely composed of underdeveloped nations with economic hardship and social instability; accordingly, careful policing of these NBC weapons is difficult, and the weapons might be obtained by international terrorists through theft or illegal purchasing.

### Problems in emergency medical preparedness and response

#### Command and control

An emergency united command system, including local personnel and military personnel, is often established temporally to command and control all emergency response teams including medical rescue teams during a NBC incident; however, these operations are often inefficiently managed because of the lack of professionals who are familiar with emergency medical rescue work. Almost no field and tabletop exercises involve the coordination of command and control between local medical services and military medical services, or trans-regional and trans-provincial coordination. Moreover, rational security priority is not endorsed for emergency medical rescue teams. Not only have security checks related to NBC agents not been established but some necessary pre-hospital inspections in many important sites have not been performed. Therefore, suspected terrorists can potentially carry out harmful operations.

#### Medical preparedness

First, responders must take necessary measures to protect themselves before entering a contaminated area to conduct medical service. Currently, all of the national medical rescue teams in China are equipped with detection equipment and protection apparatuses, but the equipment is not sufficient for real incidents, which indicates that replacements are not available if some instruments do not function. Second, most medical facilities either have insufficient quantities of antidotes or do not have any stockpiled antidotes, which would lead to a large number of casualties after a NBC incident. In addition, the criteria for the type and quantity of stockpiled antidotes have not been finalized. Finally, overall comprehensive training for NBC incidents is generally conducted according to fixed plans within military organizations without a review of local hospital and community involvement; additionally, only a single role in this exercise is performed under a given scenario without consideration of the other roles, which demonstrates that these plans and exercises for responding to NBC incidents remain divorced from reality.

#### Emergency education for NBC incidents

Courses involving NBC incidents are not available at most medical universities in China except for military medical universities; students who graduate from medical universities should have had a clear understanding of the risks of a potential NBC release, the consequences of exposure, and the correct steps that they should take to address these situations. Given the lack of knowledge of emergency medical rescue related to NBC incidents, local medical facilities other than the military medical universities might be incapable of providing medical support during a NBC incident. Additionally, the complete lack of knowledge of NBC incidents among the civilian population might lead to misunderstanding of announcements released by the government, which would thereby affect the stability of the society and lead to the incorrect use of personal protective equipment that would result in casualties even if the personal protective equipment is available.

### Countermeasures in medical rescue in response to NBC incidents

#### Enhancing effective response coordination

Proper communication between military emergency rescue units and local medical facilities is necessary in response to a NBC incident; in addition, it is still important that both systems of command and control that are initiated by military and local emergency entities maintain close communication with one another. In terms of medical rescue in response to NBC incidents, medical professionals who work for the military generally find it easier to acquire the knowledge related to NBC incidents and the treatment skills such as risk assessment of NBC incidents, first aid for victims, triage, the establishment of decontamination stations, and the management of evacuating civilians from a contaminated area. Therefore, based on proper coordination between the military and a local emergency command agency, the military medical rescue team should take more responsibility than local medical facilities and must be provided with a high level priority to perform prehospital inspection involving NBC agents; local medical facilities should focus on the surveillance of food safety and drinking water quality, lifesaving support, isolation of affected contaminated civilians, and treating victims in the hospital. Emergency rescue operations at the military, local government and state levels should be activated and carried out in an orderly manner. Moreover, it is essential that exercises for NBC incidents be conducted in parallel with a review of military, local government and community emergency plans, which will contribute to the enhanced coordination of all medical facilities.

#### Providing appropriate public education

With less knowledge of NBC incidents, the effects of social panic caused by NBC incidents are most likely worse than the actual casualties themselves [3]. To counter the spread of misinformation, it is necessary to provide public education involving NBC incidents to civilians and to keep the community well informed. Public education is more than a community activity, and school education involving NBC incidents is equally important. Regarding public education, clear dissemination of the correct steps that should be taken after the announcement of a NBC release by rescuers or the government can be achieved. Additionally, self-rescue skills, such as making good use of personal protective apparatus, and a clear understanding of the health consequences associated with NBC exposure are also indispensable and could be accomplished through community seminars on proper communicating, health publications on local radio and television, multimedia reports, and even internet-based services. In addition, education on NBC incidents could be offered in schools, as students will value and can rapidly learn some of the core elements of disaster medicine and emergency preparedness [4], especially in medical school, with information related to NBC incidents. For example, the knowledge of NBC agents can be incorporated into physics, biology, and chemistry courses.

#### Innovating practical training and exercises

Traditionally, a single role is defined in a medical rescue exercise, which results in an ineffective medical plan. The lack of the other roles in a medical rescue exercise, each of exercise schedules with no exceptions was always designed to be biased exclusively to the rescuers, and run on a given scenario ended up with managing successfully. Importantly, the other roles should be added to simulate the thoughts of terrorists regarding mounting a “real” attack involving NBC incidents, which would make the exercises or training more practical than ever before. Furthermore, training and exercise should be conducted in parallel with the CDC, as well as hospital and community plans to test the efficiency of those plans. Several evaluation methods, such as on-site surveys, a structured disaster drill evaluation tool, and a video analysis of the teamwork, are necessary to amend the overall preparedness because it cannot be adequately characterized using any one single method [5].

#### Establishing a professional consultant team or system

The rapid identification of an unknown agent can be a complicated process [6]. Even in the USA, only the FBI’s specialized assessment team contains clinicians who are experienced at identifying the signs and symptoms of exposure to chemical and biological agents [7]. Rescue operations are multidisciplinary. Thus, a specialized professional consultant team that includes physicians (primarily working in the NBC-related medical departments such as emergency medicine, microbiology, and public health), nuclear physics experts, and chemical experts who are experienced at destroying the chemical weapons abandoned in China by Japanese invaders, can be established to provide rapid support to responders in a NBC incident. Additionally, systems such as TESS (the Toxic Exposure Surveillance System, TESS) [8] should be established to collect NBC incident data to serve as proxy markers for a diagnosis and to provide alerts of potential outbreaks.

## Conclusions

Based on the consideration of the complexity and difficulty of response to NBC incidents, and the requirement of cooperation among national safety agencies, military/local/regional, health care providers, professional medical societies, military and local communities should create as early as possible a sustained and integrated response system to NBC incidents. At the national level, government needs to play an important catalyst role in encouraging its local health facility commanders to communicate with military health facility, and create a national network of commanding, training, responding, detecting, rescuing, and managing. At the educational level, public health curricula involving NBC should be available for all kinds of universities, and professional skills involving NBC should be provided for medical school. And at the exercise level, both NBC exercise with opposability and an effective evaluation are unprecedentedly necessary to response to potential NBC incidents. Finally, assistant decision-making in a NBC incident remains to be done by a professional expert group, including emergency responders, planners, nuclear physics experts, microbiological experts, chemical experts and countermeasures experts.

## Abbreviations

NBC: Nuclear, Biological and chemical
TESS: the Toxic Exposure Surveillance System
WMD: Weapons of mass destruction
